# Supplementary material for: The causal relationship between the human gut microbiota and pyogenic arthritis: a Mendelian randomization study
Source: Front Cell Infect Microbiol. 2024 Nov 26;14:1452480. doi: 10.3389/fcimb.2024.1452480 (PMC11629706; doi:10.3389/fcimb.2024.1452480)
Supplement: Supplementary file 1 [file DataSheet1.zip › mendelian test/ebi-a-GCST90027607.csv_leave_one_out.pdf]

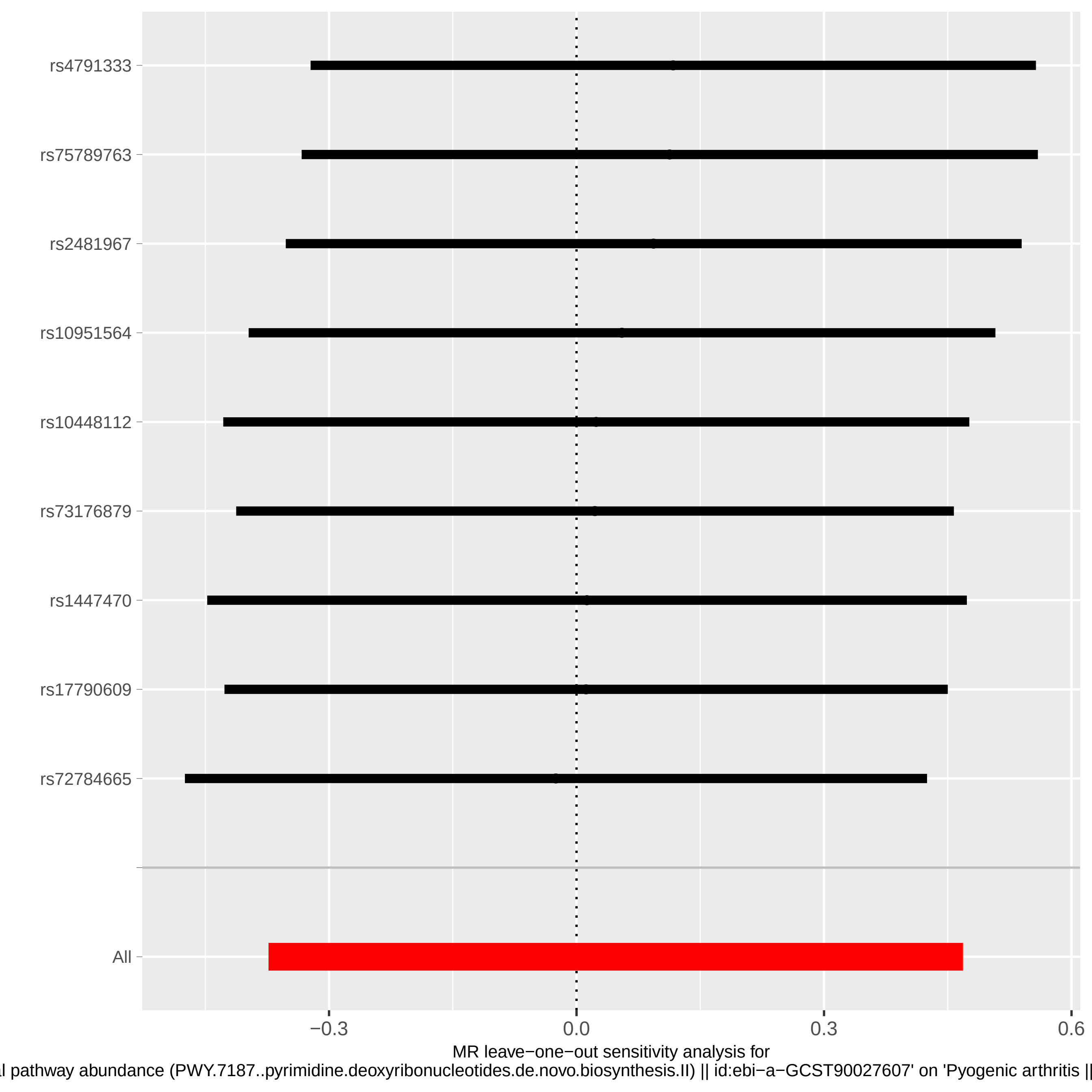
rs4791333
rs75789763
rs2481967
rs10951564
rs10448112
rs73176879
rs1447470
rs17790609
rs72784665
All
−0.3
0.0
0.3
0.6
MR leave−one−out sensitivity analysis for
l pathway abundance (PWY.7187..pyrimidine.deoxyribonucleotides.de.novo.biosynthesis.II) || id:ebi−a−GCST90027607' on 'Pyogenic arthritis ||
